# Ventral Striatal Reactivity in Compulsive Sexual Behaviors

**DOI:** 10.3389/fpsyt.2018.00546

**Published:** 2018-11-14

**Authors:** Mateusz Gola, Małgorzata Draps

**Affiliations:** ^1^Institute of Psychology, Polish Academy of Sciences, Warsaw, Poland; ^2^Swartz Center for Computational Neuroscience, Institute for Neural Computations, University of California, San Diego, San Diego, CA, United States

**Keywords:** compulsive sexual behaviors, problematic pornography use, hypersexuality, ventral striatum, nucleus accumbens

## Abstract

Compulsive Sexual Behaviors (CSB) are a reason to seek treatment. Given this reality, the number of studies on CSB has increased substantially in the last decade and the World Health Organization (WHO) included CSB in its proposal for the upcoming ICD-11. Sixty percent of the neuroimaging studies on CSB published since 2014 aimed to examine similarities and differences between brain mechanisms underlying CSB, gambling disorder, and substance use disorders. One of the crucial brain circuits involved in addiction is the reward system involving the ventral striatum (including nucleus accumbens). There are two distinct theories describing ventral striatal activity in addictions: Incentive Salience Theory (IST) and Reward Deficiency Syndrome (RDS). IST describes increased ventral striatal activations during the anticipation of addiction-related reward, while RDS describes decreased ventral striatal reactivity both during the anticipation of the reward and during the reward processing. Here, we aim to investigate how the findings on ventral striatal reactivity in CSB support each of these two addiction frameworks. For this purpose, we conducted a systematic review of neuroimaging studies on CSB available in Pubmed, EBSCO, and Google Scholar between 2005 and 2018. We found nine relevant research papers. Only four of these studies directly investigated processing of erotic cues and/or rewards and reported findings related to ventral striatum activations. Three of these studies indicate increased ventral striatal reactivity for erotic stimuli, which is consistent with IST and does not support predictions based on RDS. Therefore, the current state of this data suggest that CSB is related to increased ventral striatal reactivity during the anticipation of erotic stimuli.

## Introduction

Compulsive Sexual Behaviors (CSB) are a reason to seek treatment for both males (1–3) and females (4). The most commonly reported symptoms of CSB concern time spent viewing pornography (mainly on the Internet) and excessive masturbation (5–7). Other reported types of behaviors include risky casual sexual relations, anonymous sex, and use of paid sexual services (8).

The number of studies on CSB has increased substantially during the last decade (9, 10) and the World Health Organization (WHO) included CSB as an impulse control disorder (11) in its proposal for the upcoming ICD-11 (12). According to proposed criteria (very similar to those previously proposed by Kafka (6), we may recognize CSB Disorder if following symptoms are observed over a period of at least 6 months:

1. Excessive time spent on sexual fantasies, urges, or behaviors repeatedly interferes with other important (non-sexual) goals, activities, and obligations, i.e., pornography viewing has become a central interest in one's life, so that family duties or work obligations are neglected;
2. The subject engages repeatedly in these sexual activities in response to dysphoric emotional states, i.e., sexual activity has become a rigid strategy of mood regulation;
3. And/or in response to stressful situations; e.g., during stressful events at work;
4. Despite repeated attempts, the subject fails to control or significantly reduce these sexual activities, i.e., the subject makes numerous unsuccessful attempts to limit problematic activities, but invariably loses control over them after a couple of days;
5. The subject continues these sexual activities despite the risk of physical or emotional harm to self or to others, i.e., engaging in frequent sexual behavior despite serious consequences for relationships (e.g., break-up) or threat of job loss.

The frequency and intensity of these sexual activities lead to clinically significant personal distress or dysfunction in important aspects of life and do not result from exogenous substance use (e.g., drug abuse or medication), bipolar disorder, or paraphilia.

## Patterns of ventral striatal activations according to theoretical frameworks of addictions

One of the crucial brain circuits involved in addiction is the reward system connecting such brain structures as the ventral tegmental area (one of the principal dopamine-producing areas in the brain) with the ventral striatum, mesocortical pathways, and cerebral cortex, especially the orbitofrontal and mediofrontal cortex (13–16). Anatomically, the ventral striatum in humans and non-human primates includes the nucleus accumbens, the region between the caudate nucleus and ventral putamen to rostral internal capsule, the olfactory tubercle, and the rostrolateral portion of the anterior perforated space adjacent to the lateral olfactory tract (17, 18). However, human connectivity studies suggest that the ventral striatum includes the nucleus accumbens and a larger region of the medial caudate nucleus and rostroventral putamen (19).

The ventral striatum receives cortical input from the orbital frontal cortex and anterior cingulate cortex in addition to dopaminergic input from the midbrain. The same region projects output to the ventral pallidum and to the ventral tegmental area, which project output back to the prefrontal cortex through the medial dorsal nucleus of the thalamus. This circuit is an integral part of the cortico-basal ganglia system (19). Different nodes of this network play different roles in such aspects of reward processing as motivation and hedonic pleasure (20, 21). The ventral striatum (especially the nucleus accumbens) is probably the most extensively studied brain region in the context of reward processing (22, 23), demonstrating activation during the anticipation and receipt of different types of rewards (24, 25).

Of the many addiction theories which are of interest, here, we would like to focus on two which allow for very clear predictions about ventral striatal activation and its link to addictive behaviors: Incentive Salience Theory [IST, (26–28)] and Reward Deficiency Syndrome [RDS; (29, 30)].

The Incentive Salience Theory framework, proposed by Robinson and Berridge (28), distinguishes between two basic components of motivated behavior—“liking” and “wanting.” “Liking” is linked directly to the *experienced* value of the reward, usually carried by an unconditional stimuli such as heroine consumption; on the other hand, “wanting” is related to the *expected* value of the reward, often carried by a conditional stimuli (for example, the presence of people with whom one used to take drugs). Studies on substance and gambling addiction show that learned conditional stimuli (so called *cues*) related to addiction evoke increased responses in the ventral striatum as well as increased motivated behavior (manifested with shorter reaction times) among addicted individuals, while responses to the reward itself remain unchanged or undergo blunting over time (26, 31). Thus, according to IST, if CSB disorders share neural mechanisms with addictions, we should see an increased Blood-Oxygen-Level-Dependent (BOLD) response in the ventral striatum specifically for cues signaling erotic/sexual rewards, followed by higher motivation to obtain them (measured as shorter RTs) among individuals with CSB when compared to other cues predictive for other types of rewarding stimuli.

The Reward Deficiency Syndrome theory (29, 30) posits that individuals with addictive behaviors have a general deficit in recruiting brain reward pathways, resulting in chronic hypoactivation of these circuits and supposedly reduced pleasurable experience from rewards. Addictive behaviors, such as substance use or gambling, are consequently initiated to compensate for this reward deficiency and to stimulate the brain's reward circuitry (32). According to RDS, if a group of individuals with CSB is similar to subjects with substance and gambling addictions, than we should see decreased ventral striatal activations in the CSB group in response to the cue and during reward processing when compared to healthy controls.

Before discussing the results of published studies, it is worth mentioning that according to our understanding, IST and RDS are not contradictory, but rather complementary, approaches. It may seem counterintuitive since IST predicts increased ventral striatal activations for cues related to erotic/sexual reward, while RDS predicts decreased ventral striatal activations for such cues in the case of CSB individuals when compared to healthy controls. But for the sake of better understanding, we need to take the origins of both frameworks into account. RDS describes an inborn, genetically-determined tendency for hypoactivation of reward circuits. The RDS framework relates this innate trait to specific gene mutations, *except* for in the case of addictions, in which this tendency is related to non-specific mutations (20, 30, 33). On the other hand, IST assumes that incentive salience of some types of cues can be acquired through the regular conditioning and learning processes; however, in the case of individuals with a specific phenotype [for example, sign-trackers: animals that are more prone to fast learning of cues predictive for rewards (34, 35)], this learning process can be much faster.

Therefore, we can imagine that some individuals with the phenotype described by RDS have generalized hypoactivation for any type of rewards and their associated cues, and present with lower activations of the ventral striatum when compared to the general population. However, at the same time, these same individuals have learned that some types of stimuli or substances provide them with greater pleasure—thusly, all cues associated with these greater pleasure-inducing stimuli acquire high incentive salience, per conditioning (as described in IST). For these specific cues, this group's ventral striatum can be more activated than when compared to the general population and when compared to different types of cues. With this prediction, we aim to review available neuroimaging data on ventral striatal activations in CSB.

If CSB is more related only to IST, then we should find more studies showing increased ventral striatal activations during expectation for erotic stimuli among individuals with CSB as compared to healthy controls. If CSB is more related to the RDS, then we should see more studies which demonstrate decreased ventral striatal reactivity for any type of rewards among CSB subjects when compared to healthy controls, and possibly decreased reactivity of the ventral striatum during the expectation of the reward, as well.

## Methods

For the purpose of this review, we searched Google Scholar, Pubmed, and EBSCO databases for scientific papers published in peer-reviewed journals (excluding conference abstracts) between January 1, 2005 and February 22, 2018. We only included publications which utilized functional magnetic resonance imaging (fMRI), as we are interested in the BOLD response of the ventral striatum and included keywords such as compulsive sexual behavior, pornography, sex addiction, hypersexuality, hypersexual disorder, problematic pornography use, and internet pornography addiction. The search was performed on February 22 and February 25, 2018. We only included articles published in English. We have found nine publications which met our search criteria (Table 1), six of which specifically examined ventral striatal activations during erotic cue or erotic reward processing (36–42). Inclusion and/or exclusion of all listed publications was discussed by two judges. As the overall number of publications was nine (and seven reporting any effects related to the ventral striatum), we did not select studies based on the methods of CSB diagnosis; therefore, we describe the specific methods used for subjects classification in Table 1.

**Table 1:** Research publications on CSB or pornography use using functional resonance imaging.

**References**	**Subjects**	**Classification criteria and questionnaires**	**Aims and methods**	**Ventral striatal coordinates**	**Type of cue/reward**	**Description of results**
Miner et al. (43)	8 CSB and 8 controls; heterosexual males	Clinical interview using Structured Clinical Interview for DSM-IV, with extra section added to assess the symptoms of CSB Compulsive Sexual Behavior Inventory	Aims: investigate white matter micro-structure and behavioral inhibitory processes in men with CSB Methods: diffusion tensor imaging and Go—No Go Task	Not reported	None	This study was investigating performance in go-no go task, and there was no task related directly to cue or reward processing
Voon et al. (37)	19 CSB and 19 controls; heterosexual males	Clinical interview using CSB diagnostic criteria by Kafka Internet Sex Screening Test and extensive investigator-designed questionnaire	Aim: investigate neural correlates of cue reactivity comparing sexually explicit video cues with non-sexual cues in subjects with and without CSB Methods: passive viewing of video during functional magnetic resonance imaging	x = 18, y = 2, z = −2	Video clips of explicit sexual, erotic, non-sexual exciting, money, and neutral	Exposure to sexually explicit sexual videos in CSB compared to control subjects was associated with stronger ventral striatal response. Exposure to exciting non-sexual videos in CSB compared to control subjects was associated with weaker ventral striatal response. (Contrast: CSB > Control Subjects)
Kühn and Gallinat (38)	64 healthy heterosexual males	No CSB populations, but authors assessed time spent on pornography use and symptoms of CSB by Internet Sex Screening Test and Sexual Addiction Screening Test	Aim: investigate neural correlates associated with frequent of pornography use in a healthy population Methods: voxel-based morphometry, resting state functional connectivity, functional magnetic resonance imaging during Cue-Reactivity Task	x = 11, y = 5, z = 3 and x = −24, y = 2, *z* = 4	60 explicit sexual images and 60 non-sexual images	Negative association between reported pornography hours per week and gray matter volume in the right striatum as well as with functional activity during a sexual cue–reactivity paradigm in the left putamen. (Parametric modulation of BOLD by amount of pornography use)
Seok and Sohn (40)	23 CSB and 22 controls; heterosexual males	Clinical interview using CSB diagnostic criteria by Kafka Sexual Addiction Screening Test-R and Hypersexual Behavior Inventory	Aims: investigate sexual desire in men with CSB and identify neural correlates of enhanced desire Methods: passive viewing of images during functional magnetic resonance imaging	x = −38, y = −32, z = 2	34 images depicting naked women and sexual activity, and 20 non-sexual images	Higher activation of left caudate nucleus for erotic pictures in CSB group when compared to controls. Lower activation for neutral pictures in left caudate nucleus in CSB group when compared to controls. (Contrast: CSB > Control Subjects)
Banca et al. (42)	23 CSB and 40 controls; heterosexual males	Clinical interview using CSB diagnostic criteria by Kafka Internet Sex Screening Test and extensive investigator-designed questionnaire	Aims: investigate novelty-seeking and cue-conditioning in individuals with CSB, investigate neural correlates of cue-conditioning in men with CSB Methods: conditioning imaging task and habituation during functional magnetic resonance imaging	x = 2, y = 8, z = −10	Colored patterns as a cue and sexual, monetary, and neutral image as a reward	Decreased ventral striatal activations among CSB subjects (when compared to control) as a response for lack of erotic or monetary reward. (contrast: CSB > Control Subjects)
Brand et al. (39)	19 healthy heterosexual males	Internet Addiction Test modified for cybersex, Hypersexual Behavioral Inventory	Aim: investigate neural responses to pornographic material that is consistent with subjects' sexual preferences compared to pornographic material Methods: picture valuation task and picture choice task during functional magnetic resonance imaging	x = 14, y = 8, z = −8 and x = −8, y = 6, z = −10	Explicit sexual images in 3 categories: interactions between a man and a woman, between 2 men, and between 2 women	Increased ventral striatal activation in response to preferred vs. non-preferred erotic pictures. Positive correlations between ventral striatal activation (during presentation of erotic pictures) and score in Internet Addiction Test (s-IAT) (44) (Contrast: Preferred erotic pictures > Non-preferred erotic pictures)
Klucken et al. (41)	20 CSB and 20 control heterosexual males	Clinical interview using CSB diagnostic criteria by Kafka	Aim: investigate differences in neural activity associated with appetitive conditioning and connectivity in men with and without CSB Methods: appetitive conditioning paradigm during a functional magnetic resonance imaging	x = −15, y = −1, z = −2	2 colored squares as a cue and 21 erotic pictures as a reward	This study explores group differences in neural activity associated with appetitive conditioning and shows decreased coupling between the ventral striatum and prefrontal cortex in the CSB vs. control group. (Contrast: CSB > Control Subjects)
Gola et al. (36)	28 CSB and 24 control heterosexual males	Clinical interview using CSB diagnostic criteria by Kafka Sexual Addiction Screening Test-R and extensive investigator-designed questionnaire	Aim: investigate neural correlates of sexual and non-sexual incentives in men with and without CSB Methods: incentive delay task during functional magnetic resonance imaging	x = −12, y = 10 z = −6 and x = 12, y = 10, z = −4	Erotic, monetary, and control symbols as cues, erotic images and money as a reward	This study use incentive delay task with 2 types of rewards—erotic and monetary. Men with and without CSB differed in their striatal responses to cues predicting erotic pictures but not in their responses to erotic pictures or monetary rewards. CSB subjects when compared with control subjects showed increased activation of ventral striatum specifically for cues predicting erotic pictures but not for cues predicting monetary gains. (Contrast: CSB > Control Subjects)
Seok and Sohn (45)	17 CSB and 19 control heterosexual males	Clinical interview using CSB diagnostic criteria by Kafka Sexual Addiction Screening Test-R and Hypersexual Behavior Inventory	Aim: investigate gray matter deficits and resting- state abnormalities in individuals with CSB Methods: voxel-based morphometry, resting state functional connectivity	Not reported	None	This study was investigating gray matter deficits and resting-state connectivity in CSB

## Review of existing data on ventral striatal activations in CSB

First, we will discuss studies directly addressing cue and reward processing. Among the seven studies reporting ventral striatal activations for erotic cues or rewards, two were conducted on a sub-clinical population [frequent pornography users; (38, 39) not fulfilling criteria of CSB] and the remaining five were conducted on clinical populations fulfilling criteria of CSB [either subjects who were presenting with a variety of CSB (37, 40–42) or individuals seeking treatment specifically for problematic pornography use (36)]. Two studies were conducted on the same population (37, 42). All studies used erotic pictures, but one utilized explicit video clips (37). In Kühn and Gallinat (38), Seok and Sohn (40), and Banca et al. (42), authors compared ventral striatal reactivity between erotic and neutral pictures, in Voon et al. (37), between explicit and exciting videos, in Brand et al. (39), between preferred and non-preferred erotic pictures, and in Gola et al. (36) between erotic pictures and monetary rewards and between cues predictive for erotic pictures and for monetary gains.

## Discussion

Taking into account the very limited body of experimental publications (seven) reporting activations of ventral striatum during processing of erotic and non-erotic stimuli in populations meeting the criteria of CSB or in sub-clinical populations, deriving any strong conclusions at this moment would be premature. Therefore, we would first like to discuss the available results, then propose their interpretations in the context of IST and RDS theories.

Among non-problematic pornography users, an inverse relationship between right striatum (more precisely caudate) volume and frequency of pornography consumption was observed (38). The same study also reported a negative correlation between the amount of pornography consumption and functional reactivity of the left putamen during sexual stimuli watching. Alternatively, Voon et al. (37) showed that men meeting CSB criteria (6) as compared to those without CSB, demonstrated an increased striatal reactivity for sexually explicit videos. Interestingly, CSB patients watching exciting videos (namely, presentations of extreme sports) showed lower activations in the ventral striatum when compared to controls (37). Seok and Sohn (40) showed higher activation of the left caudate nucleus in response to erotic pictures in the CSB group when compared to controls and lower activation for neutral pictures in the left caudate nucleus. Brand et al. (39), similarly to Voon et al. (37), showed increased BOLD responses in the ventral striatum in response to preferred sexual pictures when compared to non-preferred ones, and that this activity positively correlated with scores on the Internet Addiction Test Modified for Cybersex in a sub-clinical population (39). The fifth study (36) used a different paradigm than the four previously discussed. Instead of simply presenting different types of stimuli (e.g., erotic, exciting, or neutral pictures), this study used a modification of the incentive delay task, a task previously used in studies of gambling disorder (46). This task has two important properties: (1) it disentangles cue- and reward-related phases related to anticipation and outcome, respectively, and (2) it provides a possibility to compare “addiction-related” stimuli (in this case, erotic pictures) with another potent reward (monetary gains). In this study, men with and without CSB differed in their striatal responses to cues predicting erotic pictures, but not in their responses to erotic pictures. CSB subjects when compared to control subjects showed increased activation of ventral striatum specifically for cues predicting erotic pictures, but not for cues predicting monetary gains. Relative sensitivity to cues predicting erotic pictures vs. monetary gains was significantly related to the increased behavioral motivation to view erotic images (suggestive of higher “wanting”), severity of CSB, amount of pornography use per week, and number of weekly masturbations. Excepting Kühn and Gallinat (38), the other reviewed studies suggest increased sensitivity either to erotic stimuli (37, 39) or to the cues predicting erotic stimuli (36) among people with higher scores in CSB.

Of the other studies not strictly related to cue or reward processing, Banca et al. showed decreased ventral striatal activations among CSB subjects when compared to controls as a response to lack of erotic or monetary reward in a conditioning task (42). Klucken et al. (41) showed decreased coupling between the ventral striatum and prefrontal cortex in the CSB vs. control group during appetitive conditioning [in a similar task to Banca et al. (42)].

## Conclusions

If we focus strictly on ventral striatum activity in all the above mentioned studies, then a consistent schema of results emerges: preferred erotic pictures (39), explicit videos (37), or cues predicting erotic pictures (36) evoke stronger ventral striatal activations than other types of stimuli among people with CSB (or frequent pornography users) when compared to controls. Data provided by Kühn and Gallinat (38) and collected from a non-clinical sample also suggest decreased ventral striatum volumetry among healthy individuals who use more pornography; however, recent findings (47) do not confirm this difference in ventral striatum volume between individuals meeting CSB criteria and controls. As of yet, there is no study on a population meeting CSB criteria, testing BOLD responses for erotic stimuli, and examining volumetric changes at the same time, so any speculation on the relations between striatal volumetry and reactivity would be premature at this point.

## Consistency with reward deficiency syndrome

To examine published results in the light of RDS, we need to look at the differences in ventral striatal activations between CSB (or sub-clinical populations) and control groups. RDS predicts hypoactivation for rewarding stimuli and for cues predicting such stimuli in between group comparison. None amongst the four studies examining reactivity for erotic stimuli (36–39) indicates such hypoactivation in the case of erotic stimuli. However, in Voon et al. (37), the CSB group when compared to controls presents visible hypoactivation of ventral striatum for non-erotic exciting stimuli [in Seok and Sohn (40), there is visible hypoactivation in CSB individuals when compared to controls for neutral stimuli]. Opposite results are presented in Gola et al. (36) where there is no difference in BOLD response for monetary rewards between CSB and control subjects. Three (36, 38, 39) out of four available studies speak clearly against predictions formulated based on the RDS framework. However, it is important to bear in mind the differences between the groups in these studies. While in Voon et al. (37), subjects who met CSB criteria presented a variety of problematic sexual behaviors, in Gola et al. (36) all individuals who met CSB criteria presented with problematic pornography use as a dominant problem. Similarly, in two (38, 39) other studies on sub-clinical populations, ventral striatal activations and volumetry correlated with the amount of pornography use. There is not enough data to formulate any strong conclusions, but some hypothesis for future studies can be formulated.

From our point of view, it is worth investigating whether CSB can be distinguished into two subtypes characterized by: (1) dominant interpersonal sexual behaviors, and (2) dominant solitary sexual behaviors and pornography watching (48, 49). Based on analogous findings on alcohol abuse, each of these subtypes could be related to the different genotypes and patterns of ventral striatal activations for cues and rewards (50, 51). We propose to examine in future studies whether a subtype defined by interpersonal sexual behaviors can be characterized by a higher degree of novelty seeking and ventral striatal hypoactivity as proposed by RDS, while a subtype related to predominant problematic pornography viewing and solitary sexual activity can be characterized instead by increased ventral striatal reactivity for erotic cues and rewards without hypoactivation of reward circuits.

## Consistency with incentive salience theory

According to IST, learned cues (conditional stimuli) related to addiction evoke increased responses in the ventral striatum and evoke increased motivated behavior (i.e., shorter reaction times and higher accuracy) among addicted individuals, while responses to the reward itself remain unchanged or undergo blunting over time (26, 31). Thus, according to IST, if CSB shares mechanisms with addictions, we should see an increased BOLD response in the ventral striatum specifically for cues signaling erotic/sexual rewards among individuals with CSB when compared to healthy controls and when compared to the reaction for cues predicting other rewards.

Reading each of the presented publications (36–39) separately, one might gather that all data consistently indicate mechanisms proposed by IST, namely, higher sensitization for erotic stimuli. But one very important question emerges: How to interpret these erotic stimuli in the laboratory setup? If one assumes that an erotic picture or video plays the role of cue, then increased ventral striatal reactivity among subjects with CSB (in comparison with controls) would speak in favor of the addiction hypothesis. However, if one assumes that erotic stimuli play the role of reward, then these results do not necessarily support the predictions formulated in the IST framework. From our perspective, [for details, see Gola et al. (9)] in many real life situations, visual sexual stimuli such as the naked body of a sexually attractive partner increase sexual arousal and lead to approach behaviors initializing dyadic sexual activity and ending with orgasm (52). In this case, we argue that sexual stimuli play the role of cue (conditional stimuli), while orgasm plays the role of (primary) reward (unconditional stimuli). This may be the case particularly for healthy controls and for CSB subjects with dominant interpersonal sexual behaviors.

Our reasoning is similar for most cases of solitary sexual activity, especially for healthy subjects. Most common visual sexual stimuli are pornographic videos or photos (cues), which increase sexual arousal and lead to masturbation ending with orgasm (reward). But in the research (9), we observe the following: (1) people experience pleasure while viewing erotic pictures and videos, possibly accompanied by genital reaction; (2) their rewards-related brain activity is correlated with these pleasurable feelings in response to visual sexual stimuli; (3) they are willing to exert effort to view these stimuli, similar to other rewarding stimuli, such as money; and (4) we also see conditioning for cues predictive of sexual stimuli. Thus, we claim that visual sexual stimuli may have rewarding value and, that in a laboratory setup [like in study (36)], can play the role of reward. For CSB individuals with dominant solitary behaviors and pornography watching, this may also be the case in real life situations, as many of them report pornography binges wherein orgasm is intentionally delayed to maintain hours of pleasure in pornography viewing (2). Therefore, according to our view, the results of the available studies support predictions of IST and show either increased ventral striatal reactivity for erotic stimuli [which may play the role of cue for subsequent sexual activity (37, 39)] or for cues predicting erotic pictures, which *per se* is a rewarding stimuli (36).

## Similarities to substance use and gambling disorder

Most recent meta-analysis (32) of 25 studies on ventral striatal activations in substance addictions and pathological gambling suggest that during reward anticipation (exposition to cue), individuals with substance and gambling addictions showed decreased striatal activation as compared with healthy control individuals. During reward outcome, individuals with substance addiction showed increased activation in the ventral striatum, whereas individuals with gambling addiction showed decreased activation in the dorsal striatum compared with healthy control individuals. According to the authors, striatal hypoactivation in individuals with addiction during reward anticipation and in individuals with gambling addiction during reward outcome is in line with the RDS theory of addiction. It is important to note that all of the studies included in this meta-analysis were using monetary incentives; therefore, described patterns of reactivity for cues and rewards were non-specific for certain substance related addictions. The only study with CSB subjects—which can be directly compared to the studies reviewed in Luijten et al. (32)—is Gola et al. (36), which uses the monetary incentive delay task. Here, no hypoactivation of ventral striatum in CSB (compared to controls) was observed. We see a need to conduct studies comparing CSB individuals with populations addicted to substances or gambling using standard tasks such as monetary incentive delay task to directly investigate similarities and differences between CSB and addictions in ventral striatum reactivity.

## Summary and future directions

The amount of available studies on CSB (and sub-clinical populations of frequent pornography users) is constantly increasing. Among currently available studies, we were able to find nine publications (Table 1) which utilized functional magnetic resonance imaging. Only four of these (36–39) directly investigated processing of erotic cues and/or rewards and reported findings related to ventral striatum activations. Three studies indicate increased ventral striatal reactivity for erotic stimuli (36–39) or cues predicting such stimuli (36–39). These findings are consistent with IST (28), one of the most prominent frameworks describing brain functioning in addiction. The only support for another theoretical framework which predicts hypoactivation of the ventral striatum in addiction, RDS theory (29, 30), comes partially from one study (37), where individuals with CSB presented lower ventral striatal activation for exciting stimuli when compared to controls.

The current state of the data allows us to conclude that CSB is related to increased ventral striatal reactivity for erotic stimuli and cues predictive for such stimuli. However, many basic questions allowing for direct comparisons with substance addictions and pathological gambling remain unaddressed. We see a need for studies directly comparing CSB individuals with populations addicted to substances (to verify predictions based on RDS) as well as more experimental work on cue and reward processing in CSB (for further verification of predictions based on IST). Future studies should also try to control for dominant patterns of CSB (e.g., solitary vs. interpersonal sexual activity).

We also want to note that the ventral striatum is only one brain region related to reward processing and learning, and that a much more complex picture of CSB can be presented when we are able to integrate knowledge on whole brain activity.

## Limitations

Our review has limitations related to the small number of fMRI research with CSB patients. Due to this limitation, we tried to include all studies, despite obvious differences in the diagnostic methods, and criteria they imply (see Table 1), what results with non-homogenous samples. Secondly, we took quite a broad definition of ventral striatum, including a larger region of the medial caudate nucleus and rostroventral putamen with nucleus accumbens (19). We hope that an increasing body of evidence will allow for more specific analysis in the future.

## Author contributions

All authors listed have made a substantial, direct and intellectual contribution to the work, and approved it for publication.

### Conflict of interest statement

The authors declare that the research was conducted in the absence of any commercial or financial relationships that could be construed as a potential conflict of interest.
